# Decreased Insulin Sensitivity in Telomerase-Immortalized Mesenchymal Stem Cells Affects Efficacy and Outcome of Adipogenic Differentiation *in vitro*

**DOI:** 10.3389/fcell.2021.662078

**Published:** 2021-08-04

**Authors:** Konstantin Kulebyakin, Pyotr Tyurin-Kuzmin, Anastasia Efimenko, Nikita Voloshin, Anton Kartoshkin, Maxim Karagyaur, Olga Grigorieva, Ekaterina Novoseletskaya, Veronika Sysoeva, Pavel Makarevich, Vsevolod Tkachuk

**Affiliations:** ^1^Department of Biochemistry and Molecular Medicine, Faculty of Medicine, Lomonosov Moscow State University, Moscow, Russia; ^2^Institute for Regenerative Medicine, Medical Research and Education Center, Lomonosov Moscow State University, Moscow, Russia

**Keywords:** mesenchymal stem/stromal cells, osteogenic differentiation, chondrogenic differentiation, adipogenic differentiation, insulin signaling, protein kinase A signaling

## Abstract

Modern biomedical science still experiences a significant need for easy and reliable sources of human cells. They are used to investigate pathological processes underlying disease, conduct pharmacological studies, and eventually applied as a therapeutic product in regenerative medicine. For decades, the pool of adult mesenchymal stem/stromal cells (MSCs) remains a promising source of stem and progenitor cells. Their isolation is more feasible than most other stem cells from human donors, yet they have a fair share of drawbacks. They include significant variability between donors, loss of potency, and transformation during long-term culture, which may impact the efficacy and reproducibility of research. One possible solution is a derivation of immortalized MSCs lines which receive a broader use in many medical and biological studies. In the present work, we demonstrated that in the most widely spread commercially available hTERT-immortalized MSCs cell line ASC52telo, sensitivity to hormonal stimuli was reduced, affecting their differentiation efficacy. Furthermore, we found that immortalized MSCs have impaired insulin-dependent and cAMP-dependent signaling, which impairs their adipogenic, but not osteogenic or chondrogenic, potential under experimental conditions. Our findings indicate that hTERT-immortalized MSCs may present a suboptimal choice for studies involving modeling or investigation of hormonal sensitivity.

## Introduction

Adult human mesenchymal stem/stromal cells (MSCs) were initially isolated from the bone marrow by Friedenstein et al. (1966); subsequently, MSCs have been isolated from most other tissues (Caplan and Correa, 2011). These cells play a pivotal role in maintaining normal tissue function via the support and formation of a stromal environment for tissue-specific cells. This function relies on the 1) differentiation potential of MSCs, which includes several lineages and multiple mature cell types: osteoblasts, adipocytes, chondroblasts, or fibroblasts 2) the production of tissue matrix components and paracrine regulatory factors.

This activity has also become the foundation for MSC application in regenerative medicine in its cell therapy field. Multiple experimental studies in damage models have demonstrated that MSCs participate in repair of various tissues and their recovery after injury (Qian et al., 2008; Natsumeda et al., 2017). Indications for MSC-based therapies include a wide range of conditions from bone fractures (Harris et al., 2004; Wang X. et al., 2018), cardiovascular diseases (Duran et al., 2013) to the restoration of male fertility (Sagaradze et al., 2019), and control adipose tissue function and systemic insulin sensitivity (Wang M. et al., 2018). The latter relies on the fact that MSCs are the precursors for the majority of newly formed adipocytes (Sanchez-Gurmaches and Guertin, 2014). That makes the MSCs a relevant object for both therapy and preclinical studies in light of the global spread of obesity and associated metabolic syndrome (Kopelman, 2000).

Understanding the participation of MSCs in tissue renewal, repair, and potential modulation of their physiological properties for regenerative medicine demands appropriate and relevant *in vitro* models to investigate MSC biology (Jimenez-Puerta et al., 2020). However, as an object in biomedical research, cultured primary MSCs show certain disadvantages: donor-to-donor variability and limitations during large-scale expansion which are accompanied by the fact that MSCs isolated from different tissues also exhibit significant variability (Elahi et al., 2016). A possible solution of listed problems is application of immortalized lines derived from primary human MSCs. These cells occupy a good intermediate position between highly variable primary cell cultures and lines derived from tumor cells characterized by significantly altered physiology.

One of the methods to obtain an immortalized cell line is introducing the gene encoding telomerase (TERT), an enzyme that provides restoration for telomere regions of chromosomes and thereby increases the number of possible cell divisions (Bodnar et al., 1998). Lines of hTERT-immortalized MSCs have recently become spread in biological and medical research as a substitute for primary MSCs. They were used in studies of MSC function in maintaining tissue homeostasis (Pitrone et al., 2017; Maj et al., 2018) and to create scaffolds for tissue engineering (Zitnay et al., 2018). In most studies using hTERT-immortalized MSCs, authors assumed them to be generally similar to primary human MSCs. At the same time, despite the active introduction of hTERT-immortalized MSCs in research few studies focus on functional similarities and differences between hTERT-MSC and primary MSCs and how these differences may affect the outcome of experimental studies.

We have shown that hTERT-MSC exhibited altered hormonal sensitivity compared to primary MSC culture obtained from healthy donors: in particular, they have significantly reduced sensitivity to noradrenaline (Tyurin-Kuzmin et al., 2018). As far as the sensitivity of MSCs to hormones plays a decisive role in control of their differentiation, the question of how differentiation properties of MSCs change when they are immortalized is yet to be answered. In the present work, we compared the phenotype and functional properties of hTERT-MSC and primary MSCs, focusing on differentiation to “classical” (adipogenic, chondrogenic, and osteogenic) directions.

## Materials and Methods

### Cell Cultures

hTERT-immortalized, adipose-derived mesenchymal stem cells (ASC52telo, ATCC^®^ SCRC-4,000^TM^) were maintained in the medium supporting the growth of undifferentiated mesenchymal progenitor cells (Advance Stem Cell Basal Medium; HyClone, Logan, UT, United States) containing 10% of supplement (Advance Stem Cell Growth Supplement, HyClone) and 100 U/ml of penicillin/streptomycin (Gibco; Thermo Fisher Scientific, Waltham, MA, United States). The medium was changed every 2–3 days. Cells were passaged at ≈80% confluency. All experiments were performed with cells from 15 to 25 passages.

Primary cells used in the presented study were obtained from four donors who gave their informed consent. The local ethics committee of the Medical Research and Education Center of Lomonosov Moscow State University (Moscow, Russia) approved the study protocol (#4, 04.06.2018). All donors were younger than 55, with BMI ≤ 25. Subcutaneous adipose tissue samples (0.5–5 ml) harvested during surgery were homogenized and digested in collagenase I (200 U/ml; Worthington Biochemical; Lakewood, NJ, United States) and dispase (40 U/ml; Sigma-Aldrich, St. Louis, MO, United States) solution under agitation for 30–40 min at 37°C. The tissue was then centrifuged at 200 × *g* for 10 min, and the supernatant was discarded. The pellet containing ADSC was lysed to destroy erythrocytes, filtered through a sieve (BD Falcon Cell Strainer, 100 μm; BD, Franklin Lakes, NJ, United States), and centrifuged at 200 × *g* for 10 min. The final pellet was resuspended in a culture medium. Cells were cultured in the medium supporting the growth of undifferentiated mesenchymal progenitor cells (Advance Stem Cell Basal Medium, HyClone) containing 10% of supplement (Advance Stem Cell Growth Supplement, HyClone), 100 U/ml of penicillin/streptomycin (Gibco), and 0.292 mg/ml L-glutamine (Pen Strep Glutamine, Gibco) at 37°C in a 5% CO_2_ incubator. The medium was changed every 3–4 days. Cells were passaged at 80% confluence using a HyQTase solution (HyClone). All experiments were performed with cells within five passages. MSC quantity and viability were assessed using the Countess cell counter (Invitrogen; Thermo Fisher Scientific). Proliferation activity for both cell lines was compared in the automated IncuCyte^®^ ZOOM Live Cell Analysis System (Essen Bioscience, Ann Arbor, MI, United States).

### Cell Proliferation Assay

Adipose tissue-derived MSCs of the first passage were placed in a six-well plate (100,000 cells/well). The plate was placed in an automated IncuCyte^®^ ZOOM Live Cell Analysis System (Essen Bioscience) for *in vitro* imaging of the cell culture. The survey was carried out at a frequency of once per hour for 96 h (16 fields of view/well). The device’s built-in software makes it possible to estimate the area occupied by cells by applying a “mask” to the images obtained and thus calculate the percentage of cell culture confluence. An increase in confluence directly correlates with an increase in the number of cells, making it possible to evaluate the growth rate of a cell culture by the calculated parameters.

### Flow Cytometry

MSC immunophenotype was analyzed using flow cytometry. After medium harvesting, cells were detached from culture dishes using the Versene solution and stained with antibodies against CD73, CD90, and CD105 (MSC Phenotyping Kit; Miltenyi Biotec, Auburn, CA, United States), as described in the manufacturer’s protocol. IgGs of appropriate isotype were used as a negative control. Stained cells were analyzed using FACS ARIA III cell sorter (BD).

For analysis of expression of the insulin receptor, cells were stained with antibodies to INSR alpha subunit (Thermo Fisher Scientific).

### MSC Differentiation Assays

The ability of MSC to differentiate to osteogenic, adipogenic, and chondrogenic lineages was tested *in vitro* using standard differentiation and analysis protocols described elsewhere. Briefly, cells were cultured in 12-well culture plates up to 100% confluence in all differentiation experiments. Adipogenic differentiation was induced by incubation in the growth medium based on DMEM with low glucose (HyClone) containing 10% of FBS (HyClone), 1 μMdexamethasone, 200 μM insulin, and 0.5 mM 3-isobutyl-1-methylxanthine (Millipore, Billerica, MA, United States) for 21 days. The medium was changed every 2 days. Alternatively, the adipogenic differentiation medium contained 1 mkM dexamethasone, 200 μM insulin, 0.5 mM 3-isobutyl-1-methylxanthine, and 100 μM indomethacin—agonist of PPARγ (Millipore) (Puhl et al., 2015) for 21 days. The medium was changed every 2 days.

Osteogenic differentiation was induced by incubating MSCs on collagen- and vitronectin-covered plates in a growth medium containing StemPro Osteogenesis Differentiation Kit (Thermo Fisher Scientific) for up to 21 days. The medium was changed every 2 days. Alternatively, osteogenic differentiation was induced by incubation in the growth medium based on DMEM with low glucose (HyClone) containing 10% of FBS (HyClone) and 10 μM dexamethasone + 0.2 mM ascorbic acid + 10 mM of glycerol-2-phosphate (Millipore) for up to 21 days. The medium was changed every 2 days. Differentiation efficiency was analyzed using Alizarin Red S staining. Chondrogenic differentiation was induced by incubating pelleted MSCs in StemPro Chondrogenic Differentiation Medium (Thermo Fisher Scientific) for 28 days. The medium was changed every 2 days.

### Staining of Differentiated Cultures

Cell cultures were stained with Oil Red O (Millipore) to determine the efficiency of adipogenic differentiation. Cells were fixed with 4% paraformaldehyde for 30 min. They were then stained with 0.4% Oil Red O in 60% isopropanol for 1 h.

Alizarin Red was used to estimate the efficiency of osteogenic differentiation. Cells were fixed with 10% paraformaldehyde for 30 min. They were then stained using the Osteogenesis Quantitation Kit (Millipore) following the manufacturer’s instructions.

Toluidine blue staining was used to visualize chondrogenic differentiation. Cells were fixed with 4% paraformaldehyde for 30 min. They were then stained with 0.4% Toluidine Blue in 0.2 M acetate buffer at pH 4.0.

Obtained samples were analyzed via Leica AF6000 microscope with a DFC 420C camera. To evaluate the effectiveness of adipogenic differentiation, we analyzed the percentage of cells bearing lipid droplets to the total number of cells in 12 independent fields of view for each experiment using the ImageJ software (NIH, Bethesda, MD, United States).

### RT-PCR

The RNeasy Mini Kit (Qiagen) was used to extract RNA. cDNA was synthesized from 500 ng RNA with the MMLV Reverse Transcription Kit (Evrogen, Moscow, Russia) according to the manufacturer’s instructions. The relative expression of gene-markers of osteogenic (RUNX2, osteocalcin) and adipogenic (PPARγ, adiponectin) differentiation was analyzed by quantitative real-time PCR. The following equipment were used: qPCR mix-HS SYBR + LowROX (Evrogen) reagents and CFX96 Touch Real-Time PCR Detection System (Bio-Rad, Hercules, CA, United States). The gene of 60S Ribosomal protein P0 (RPL0) was used as a housekeeping gene. Quantification and normalization of expression levels of the target genes and the reference gene (RPLP0) were calculated using the comparative threshold cycle (CT) method. Primers for PCR were picked using the NCBI Primer Designing Tool. Primer sequences are presented in the Table 1.

**TABLE 1:** List of primers used in the present work.

Target	Gene	Primers	Amplicon size (bp)
60S Ribosomal protein P0	RPL0	F: GCTGCTGCCCGTGCTGGTG R: TGGTGCCCCTGGAGATTTTAGTGG	130
Adiponectin	ADIPOQ	F: GACCAGGAAACCACGACTCA R: TTTCACCGATGTCTCCCTTAGG	199
Peroxisome proliferator-activated receptor gamma	PPARγ	F: TCAGGTTTGGGCGGATGC R: TCAGCGGGAAGGACTTTATGTATG	147
Runt-related transcription factor 2	RUNX2	F: TCTTAGAACAAATTCTGCCCTTT R: TGCTTTGGTCTTGAAATCACA	136
Osteocalcin	OCN	F: AGCAAAGGTGCAGCCTTTGT R: GCGCCTGGGTCTCTTCACT	63

### Gel Electrophoresis and Western Blotting

Cell protein samples were obtained via cell lysis in a sample buffer (62.5 mM Tris-HCl pH 6.8, 2.5% SDS, 0.002% Bromophenol Blue, 5% β-mercaptoethanol, 10% glycerol). Proteins were divided by the SDS-PAGE method. Afterward, proteins were transferred from polyacrylamide gel to the PVDF membrane by Western blotting. TBS containing 0.1% Tween-20 and 5% BSA (PanEco, Moscow, Russia) was used to prevent non-specific binding. The next step was overnight staining of the membrane with protein-specific antibodies to phosphorylated T308 Akt [Anti p-Akt (Thr308) (244F9) Rabbit mAb #4056; Cell Signaling Technology Inc., Danvers, MA, United States] and Vinculin (Anti Vinculin Rabbit antibody V4139; Sigma-Aldrich). Unbound antibodies were then washed away, and the rest were incubated with antibodies for total rabbit immunoglobulins conjugated with peroxidase (P-GAR Iss; IMTEK, Moscow, Russia) for 1 h. Amplified chemiluminescence was used as a visualization method with a Clarity ECL detection kit (Bio-Rad). Image registration was carried out using the ChemiDoc Touch gel documenting system (Bio-Rad). Image analysis and volume measurements were performed using the Image Lab Software (Bio-Rad). Total Akt staining volume readings were normalized to the respective vinculin level, and then volume readings for p-Akt were compared with respective normalized Akt.

### Registration of PKA Activity Using PKA-Spark Sensor

To deliver the cAMP sensor to hTERT-MSCs, the lenti-PKA-SPARK construct was used as described earlier (Tyurin-Kuzmin et al., 2020). Lentiviral particles (LVPs) were assembled in HEK293T cells using the standard PEI transfection protocol (Longo et al., 2013). The conditioned medium containing LVPs was collected 48–72 h post-transfection and separated from cell debris by centrifugation at 4,000 × *g*, 4°C for 15 min. Protamine sulfate (50 μg/ml) was added to the medium containing LVPs to increase transduction efficiency. Cells were cultured on 24-well plates, incubated in the medium containing LVPs, and subjected to centrifugation at 800 × *g* RT for 1.5 h to assist virus entry. After centrifugation, the medium was changed to the standard culture medium, and cells were incubated for ∼8 h. The transduction procedure was repeated two times. Between transductions, the virus stock was stored at + 4°C.

To register PKA activation with PKA-Spark, we grew transduced cells in 24- or 48-well plates at low densities to prevent cell-to-cell communications during imaging of signaling. Before the experiment, the growth medium was changed to Hanks Balanced Salt Solution (PanEco) with 20 mM Hepes (HyClone). Cells were treated with 10^–6^ M of forskolin (Abcam, Cambridge, United Kingdom) or 10^–4^ M of direct activator of protein kinase A 6-Bnz-cAMP (Biolog, Bremen, Germany) after registration of the basal activity of the cells. Activation of PKA was measured in individual cells using an inverted fluorescent microscope Nikon Eclipse Ti equipped with an objective CFI Plan Fluor DLL 10 × /0.3 (Nikon, Tokyo, Japan) and digital cooled monochrome CCD camera Nikon DS-Qi1 (Nikon). We used the simultaneous measuring of 6 × 6 fields of view in Large Image mode to increase the number of analyzed cells. Movies were analyzed using NIS-Elements (Nikon) and ImageJ software (NIH).

### Statistical Data Analysis

Experimental data were expressed as means ± standard deviation (SD). Because of the size of experimental groups, we used the Mann–Whitney *U* test, which was performed with the Statistica 6.0 software (StatSoft, Tulsa, OK, United States), and statistical significance was accepted at *p* < 0.05.

## Results

### hTERT-MSCs Demonstrate Immunophenotype Similar to Primary MSC and a Higher Proliferation Rate

**FIGURE 1:**
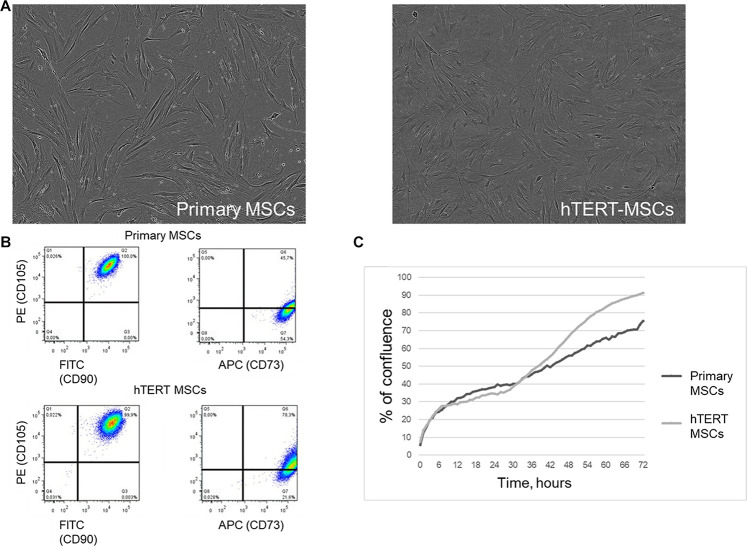
Human adipose-derived primary MSCs and hTERT-MSCs share similar morphology and immunophenotype characteristics of multipotent MSCs. **(A)** Morphology of cultivated MSCs and hTERT-MSCs, phase contrast microscopy, magn. ×200. **(B)** Evaluation of MSC markers by flow cytometry. More than 99% of MSCs express CD73, CD90, and CD105. **(C)** Proliferation curves of primary MSCs and hTERT-MSCs over a 72-h time-lapse in IncucyteZOOM. Presented data refer to the *n* = 8 independent experiments on primary MSCs from four different donors.

Both adipose-derived primary MSCs and hTERT-MSCs showed spindle-like shape morphology (Figure 1A) in culture and shared a similar immunophenotype characteristic for multipotent MSC (Dominici et al., 2006; Figure 1B). As expected, hTERT-MSCs had significantly higher proliferation activity than primary MSCs: from 1.25- up to a 3-fold decrease of population doubling time (PDT). Still, the growth curve shapes were comparable for both studied cell lines (Figure 1C).

### hTERT-MSCs Demonstrate Reduced Adipogenic, but Not Osteogenic or Chondrogenic Potential Compared to Primary MSC

**FIGURE 2:**
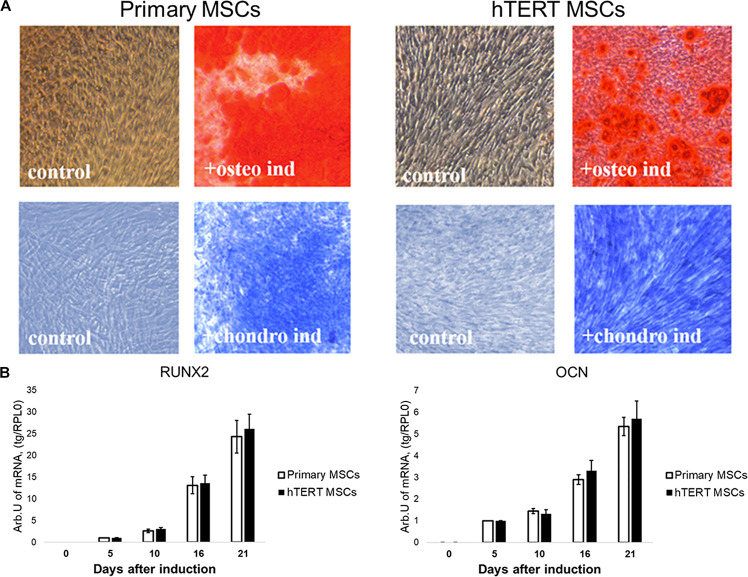
Human adipose-derived primary MSCs and hTERT-MSCs show comparable osteogenic and chondrogenic differentiation potential. Both studied MSCs differentiated into osteocytes (Alizarin Red S staining, “osteo ind”) and chondrocytes (Toluidine Blue staining, “chondro ind”) under inductive conditions. Groups “control” related to the cells cultured in standard conditions without differentiation induction. **(A)** Representative images, phase contrast microscopy, 10× objective. **(B)** Temporal dynamics of marker gene expression during differentiation in MSCs and hTERT-MSC; RT-PCR. Results are presented as mean ± SD, *n* = 6 independent experiments on primary MSCs from three different donors analyzed with Mann–Whitney *U* test.

Both cell lines were effectively differentiated into osteogenic and chondrogenic lineages under specific inductive conditions (Figure 2A). There were no detectable differences between primary MSCs and hTERT-MSCs supported by similar dynamics of respective marker gene expression in both cell lines (Figure 2B).

**FIGURE 3:**
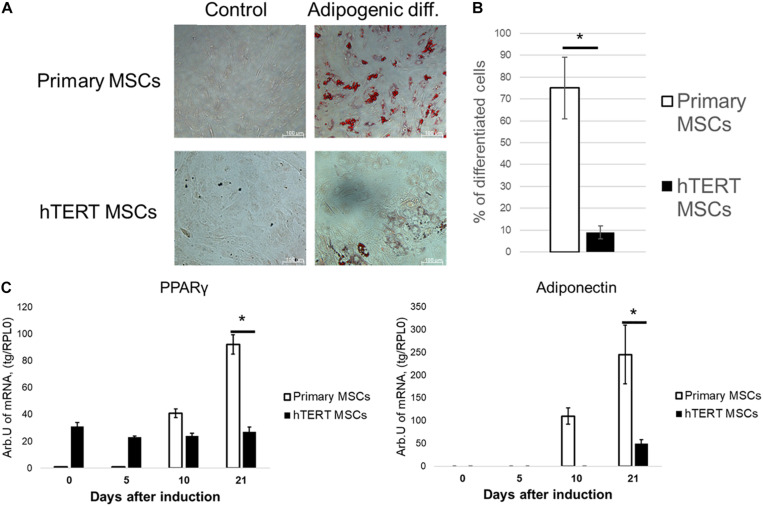
Human adipose-derived primary MSCs and hTERT-MSCs demonstrate significant differences in adipogenic potential. MSC differentiated into adipocytes (Oil Red O staining, “Adipogenic diff.”) under inductive conditions. Groups “control” related to the cells cultured in standard conditions without differentiation induction. **(A)** Representative images, phase-contrast microscopy. **(B)** Relative efficiency of adipogenic differentiation of primary MSCs and hTERT-MSCs. **(C)** Temporal dynamic of marker gene expression during differentiation of MSCs and hTERT-MSC. Results are presented as mean ± SD, *n* = 8 independent experiments on primary MSCs from four different donors. *Mann–Whitney *U* test, *p* < 0.05.

However, when subjected to adipogenic inductive conditions, hTERT-MSCs demonstrated a significantly impaired ability to differentiate into adipocytes compared to primary MSC. After 21 days of induction, hTERT-MSCs contained <10% of morphologically differentiated adipocytes (Figures 3A,B), whereas in primary MSC culture >65% of cells already accumulated lipid droplets. Marker gene expression concordantly showed a dramatic difference between hTERT-MSCs and primary MSC (Figure 3C). The latter demonstrated a significant stable increase in expression of both PPARγ (a master-gene of adipogenic differentiation) and adiponectin (an adipokine upregulated in several orders of magnitude during differentiation to adipocytes). In hTERT-MSCs, PPARγ expression remained relatively stable throughout the differentiation timeline. However, it was significantly lower than in primary MSC at Day 21 of the experiment despite increased baseline (Day 0) expression vs. primary MSC without induction of adipogenesis. At day 21, adiponectin expression in hTERT-MSCs was also significantly lower than in primary MSCs.

Overall, comparison of differentiation potential in primary and immortalized MSCs suggested that hTERT-based immortalization of these cells impaired their potential for adipogenic, but not osteogenic or chondrogenic, differentiation under conventional inductive conditions.

### In hTERT-MSC PPARγ Agonist, Indomethacin Restores Their Ability for Adipogenic Differentiation

Observed decline of adipogenic potential in hTERT-MSC may result from two putative factors: (1) decreased number of cells capable for adipogenic differentiation or (2) disrupted hormonal sensitivity, which reduced cells’ sensitivity to factors inducing adipogenesis.

To address the first hypothesis, cells were subjected to adipogenic differentiation in the presence of indomethacin—a direct PPARγ agonist (Puhl et al., 2015) able to stimulate adipogenesis bypassing hormonal signaling pathways that regulate it under physiological conditions (Cristancho and Lazar, 2011).

**FIGURE 4:**
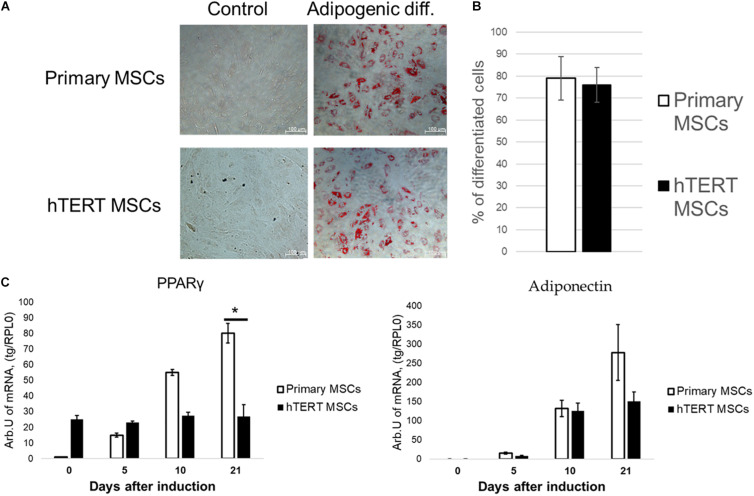
Indomethacin reduces differences in adipogenic potential between human adipose-derived primary MSCs and hTERT-MSCs. MSC differentiated into adipocytes (Oil Red O staining, “Adipogenic diff.”) under inductive conditions in the presence of indomethacin. Group “control” related to the cells cultured in standard conditions without differentiation induction. **(A)** Representative images, phase-contrast microscopy. **(B)** Relative efficiency of adipogenic differentiation of primary MSCs and hTERT-MSCs. **(C)** Temporal dynamic of marker gene expression during differentiation in MSCs and hTERT-MSC. Results are presented as mean ± SD, *n* = 8 independent experiments on primary MSCs from four different donors. *Mann–Whitney *U* test, *p* < 0.05.

As seen in Figures 4A,B, indomethacin effectively abolished differences between hTERT-MSC and primary MSC in adipogenic differentiation. After 21 days of differentiation, both cell lines demonstrated similar lipid droplet accumulation and the percentage of differentiated cells (Figure 4B). While the dynamics of adiponectin expression became similar between primary and hTERT-immortalized MSCs, PPARγ expression still differed dramatically between cell lines (Figure 4C). These results correspond with indomethacin mechanism of action. It has been shown that direct PPARγ activators (such as indomethacin and rosiglitazone), while promoting the expression of PPARγ targets such as adiponectin, did not increase expression of this transcription factor itself (Muhlhausler et al., 2009). An increase in PPARγ expression requires the activation of the classical insulin/PI3K/Akt signaling pathway (Rieusset et al., 1999). This suggests that disrupted hormonal sensitivity was a more probable cause of differences in adipogenesis in these cell lines.

### Insulin-Dependent and cAMP-Dependent Signaling Are Impaired in hTERT-MSC

To address the hormonal sensitivity of hTERT-MSCs, we evaluated the activity of two major adipogenic pathways in these cells, namely, insulin-dependent and cAMP-dependent signaling. Insulin is a primary adipogenic hormone *in vivo* and an indispensable component in most differentiation protocols (Accili and Taylor, 1991; Cristancho and Lazar, 2011; Scott et al., 2011).

**FIGURE 5:**
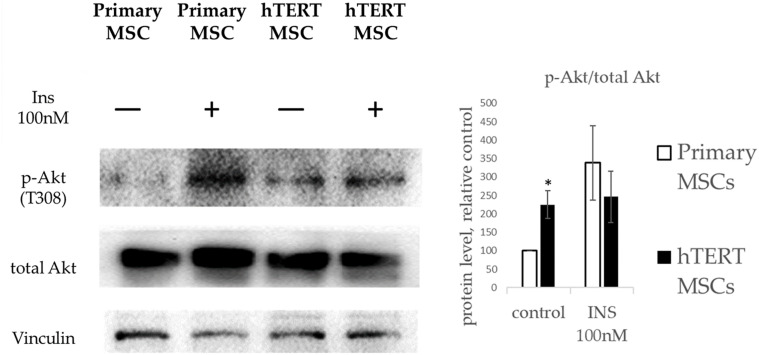
Insulin-dependent Akt phosphorylation in primary MSC and hTERT-MSC. P-Akt levels in cells are normalized to p-Akt levels in untreated primary MSCs (control). Results are presented as mean ± SD, *n* = 4 independent experiments on primary MSCs from four different donors. *Mann–Whitney *U* test, *p* < 0.05.

We found no differences in expression of insulin receptors between primary MSC and hTERT MSC (Supplementary Figure 1). As seen from Figure 5, primary MSCs responded to insulin by increased Akt phosphorylation. At the same time, hTERT-MSCs failed to react similarly after treatment by insulin in the same concentration. Furthermore, hTERT-MSCs demonstrated a significantly higher basal level of phosphorylated Akt. Therefore, the lack of p-AKT increase in response to a respective hormonal stimulus in hTERT-MSCs can be described as insulin resistance. Interestingly, in hTERT-MSC pattern of Akt activation corresponded with PPARγ expression—a high basal level was accompanied by the lack of upregulation after insulin treatment. In light of Akt being a crucial activator of PPARγ expression (Peng et al., 2003), this may explain the inability of hTERT-MSC to upregulate PPARγ expression during differentiation (Figures 3C, 4C).

**FIGURE 6:**
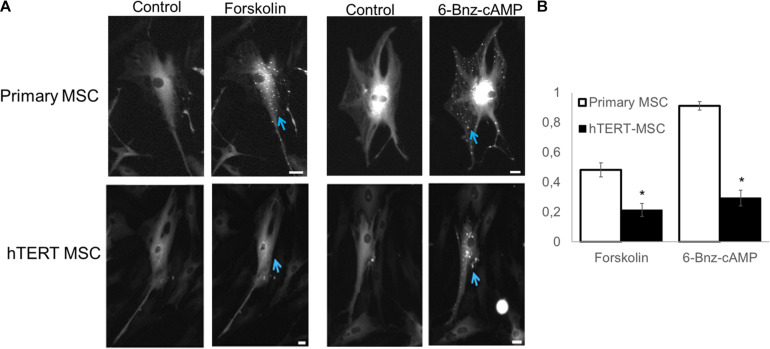
Detection of PKA activation using PKA-Spark sensor**. (A)** Representative images of sensor response to different stimuli. **(B)** Comparing the percentage of responsive cells in primary and hTERT-immortalized MSC culture. Results are presented as mean ± SD, *n* = 4 independent experiments on primary MSCs from two different donors. *Mann–Whitney *U* test, *p* < 0.05.

Another significant component in the medium used for adipogenic differentiation is IBMX (3-isobutyl-1-methylxanthine), which inhibits cellular phosphodiesterase and increases cAMP concentration (Parsons et al., 1988), resulting in activation of PKA. We used the PKA-Spark sensor to evaluate the status of cAMP-dependent signaling in hTERT-MSCs Figure 6A.

As seen from Figure 6B, only a small percentage of cells in hTERT-MSCs (solid black bars) can respond via the cAMP-dependent pathway resulting in PKA activation. For example, a direct activator of adenylyl cyclase forskolin invoked response in 21% ± 4.3% of cells, and an activator of PKA 6-Bnz-cAMP leads to response of 29% ± 5.2% of cells in the population. In contrast primary, MSCs (white bars on Figure 6B) showed 48.2% ± 4.7% sensitivity to forskolin and 91.2% ± 2.8% to 6-Bnz-cAMP as we reported earlier (Tyurin-Kuzmin et al., 2020).

Presented data suggest that in hTERT-MSCs both insulin-dependent signaling and cAMP-dependent signaling are significantly impaired, which explains their reduced ability for adipogenesis after stimulation by hormones in particularly, insulin—a major regulator of metabolism and adipose tissue turnover.

## Discussion

Immortalized MSCs are becoming widespread as a model object for fundamental research in stem cell biology, tissue engineering, and regenerative biology (Nardone et al., 2017; Maj et al., 2018; Sim et al., 2018; Zitnay et al., 2018). From a practical perspective, they possess many features that allow their reproducible and feasible use in research. Immortalized MSCs are devoid of disadvantages existing in primary MSCs: variability between cell samples obtained from different donors, limited ability to retain their physiological properties during passage in culture, and well-known resistance to most non-viral gene delivery approaches which limits their modification. This makes immortalized MSCs a promising object for practical applications in tissue engineering and a potentially valuable object for experimental models that can be used to establish molecular mechanisms of tissue renewal, particularly the differentiation of adult multipotent cells.

Notably, accumulating evidence supports the idea that MSCs could be a promising tool for cell-based therapy, primarily due to their multipotency, anti-inflammatory, and immunomodulatory properties as well as interactions with their immediate surroundings to provide regenerative cell-based responses. Furthermore, the medical utility of MSCs continues to be investigated in more than 1,000 clinical trials with a broad spectrum of diseases (reviewed in detail in Pittenger et al., 2019). Immortalized MSC advantages suggest they would benefit translational studies and overcome limitations associated with primary MSCs (Piñeiro-Ramil et al., 2019). Specifically, these cells might be preferred for studies investigating the therapeutic potential of MSC secretome. Thus, we recently reported that the extracellular matrix, produced by hTERT-immortalized MSCs, could potentiate the sensitivity of the primary stem cells to differentiation stimuli and hold a promising option to functionalize biomaterials for tissue engineering (Novoseletskaya et al., 2020).

In this work we found that the most widely commercially available immortalized MSC cell line ASC52telo shows significant alteration of differentiation potential compared to primary adipose-derived MSCs. While retaining osteogenic and chondrogenic differentiation in hTERT-MSCs comparable to primary cells, their ability to differentiate to adipocytes was significantly reduced (Figures 3A,B,C). This is probably an outcome of the TERT-based immortalization procedure. Addition of indomethacin, a direct activator of an adipogenic master regulator (PPARγ), restored hTERT-MSCs adipogenic potential (Figures 4A,B,C) indicating that hTERT-MSCs possess an ability for adipogenic differentiation, which corresponds with previous studies of hTERT-MSC characteristics in which indomethacin was used as an additional stimulator of adipogenic differentiation (Wolbank et al., 2009).

We also found that in hTERT-MSCs, two pivotal signaling cascades determining adipogenic differentiation, namely, insulin signaling and cAMP-dependent cascade, are markedly impaired. Western blotting analysis showed that hTERT-MSCs have significantly upregulated the basal level of Akt phosphorylation and were unable to increase Akt phosphorylation in response to insulin. The latter presents a phenomenon generally observed in insulin-resistant cells (Liu et al., 2009). This abnormal Akt phosphorylation pattern may explain the differences in adipogenic differentiation between primary MSCs and hTERT-MSC. Usually, during adipogenesis, insulin upregulates PPARγ through Akt, which mediates the expression of a variety of adipogenic genes (Rieusset et al., 1999; Peng et al., 2003; Zhang et al., 2009; Martinez et al., 2020). We propose that, in hTERT-MSCs, this process is severely dysregulated, resulting in a lack of adipogenesis in insulin-based differentiation protocol. In presence of direct PPARγ activator (indomethacin) however, hTERT-MSC can undergo adipogenesis, bypassing the required activation of PPARγ by insulin. However, this process is not accompanied by canonical upregulation of PPARγ expression but only by increased expression of PPARγ-regulated genes. This corresponds with described effect of direct PPARγ activators on its own expression (Muhlhausler et al., 2009).

Using a genetically encoded PKA activity sensor, we found that the cAMP-dependent signaling pathway is also significantly disrupted in hTERT-MSCs. Typically, all cells in the total population of primary MSCs bear functional PKA, activated by 6-Bnz-cAMP treatment (Tyurin-Kuzmin et al., 2020). However, in hTERT-MSCs, 6-Bnz-cAMP induces a specific response only in 30% of cells. Using forskolin, a direct activator of adenylyl-cyclase, we observed that in primary MSCs, 50% of cells carry a functional adenylyl-cyclase, while in hTERT-MSC, less than 25% of the population. Suggesting the decreased ability of hTERT-MSC to respond to cAMP-dependent hormonal stimuli.

Dysregulation of hormonal signaling may explain why adipogenic differentiation of MSC was exclusively affected by immortalization. The majority of commonly used protocols for osteogenic or chondrogenic differentiation relies not on activation of membrane receptors and downstream signaling pathways but, on the application of steroid hormones which activate transcription factors and promote extracellular matrix production via the addition of direct activators of key enzymes (e.g., the addition of ascorbic acid for prolyl-hydroxylase). To some extent, this may be expanded to the induction of adipogenesis. Indeed, using a protocol with direct activation of transcription factor PPARγ by indomethacin, we effectively differentiated both primary and hTERT-immortalized MSCs. However, a “more physiological” approach with hormonal stimulation by insulin has proven ineffective for hTERT-MSC.

The decrease in the hormonal sensitivity of MSCs after immortalization may be related to the procedure used to establish the ASC52Telo line. To do such, primary adipose tissue MSCs have been subject to retroviral transduction to deliver the hTERT gene with subsequent clonal cell selection by an antibiotic-like compound G418 (Wolbank et al., 2009). In recent years, the heterogeneity of MSCs and their role in their functional activity have been actively discussed. Moreover, published data describe specific populations of MSCs that take the regulatory role and control differentiation processes in surrounding cells (Schwalie et al., 2018).

Our group has previously described distinct subpopulations of MSCs with dramatic differences in hormonal sensitivity and associated changes of functional activity, including adipogenic differentiation (Sysoeva et al., 2017; Ageeva et al., 2018). Furthermore, during selection whithin heterogeneous cell populations, only clones with high proliferative potential gain advantage resulting in marginal elimination of subpopulations with slower proliferation (Shakiba et al., 2019). In case of hTERT-immortalized MSC, this may have resulted in loss of MSC subpopulations that determine hormonal sensitivity and differentiation of surrounding cells. Thus, despite their convenience hTERT-MSC as a tool to investigate certain aspects of MSCs physiology, should be used with caution to evaluate hormonal signaling and control of differentiation.

## Data Availability Statement

The original contributions presented in the study are included in the article/Supplementary Material, further inquiries can be directed to the corresponding author/s.

## Ethics Statement

The studies involving human participants were reviewed and approved by the local ethics committee of the Medical Research and Education Center of Lomonosov Moscow State University (Moscow, Russia). The patients/participants provided their written informed consent to participate in this study.

## Author Contributions

KK, PT-K, and AE: Conceptualization. KK, PT-K, AE, OG, EN, and VS: Data curation. KK, PM, and VT: Funding acquisition. KK, PT-K, NV, AK, and EN: Investigation. MK, OG, and VS: Methodology. KK, PT-K, AE, PM, and VT: Writing. All authors contributed to the article and approved the submitted version.

## Conflict of Interest

The authors declare that the research was conducted in the absence of any commercial or financial relationships that could be construed as a potential conflict of interest.

## Publisher’s Note

All claims expressed in this article are solely those of the authors and do not necessarily represent those of their affiliated organizations, or those of the publisher, the editors and the reviewers. Any product that may be evaluated in this article, or claim that may be made by its manufacturer, is not guaranteed or endorsed by the publisher.

## Abbreviations

MSC: Mesenchymal stem/stromal cells
hTERT: Human telomerase reverse transcriptase
IBMX: 3-isobutyl-1-methylxanthine
PKA: Protein kinase A.
