# Supplementary figures and images for: Decreased Insulin Sensitivity in Telomerase-Immortalized Mesenchymal Stem Cells Affects Efficacy and Outcome of Adipogenic Differentiation in vitro
Source: Front Cell Dev Biol. 2021 Aug 4;9:662078. doi: 10.3389/fcell.2021.662078 (PMC8371914; doi:10.3389/fcell.2021.662078)

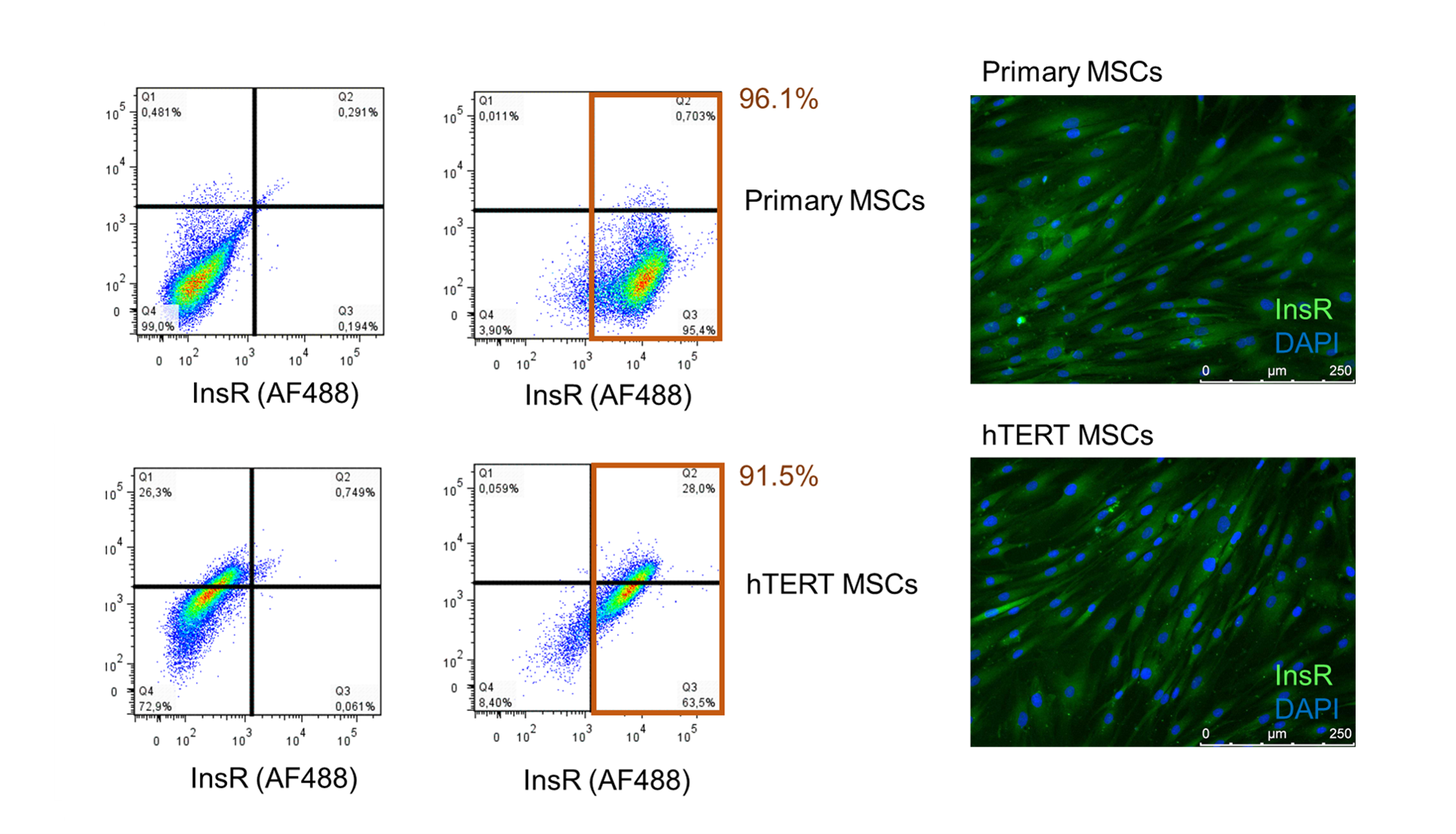

Supplement: Supplementary file 1 [file Image_1.TIF]

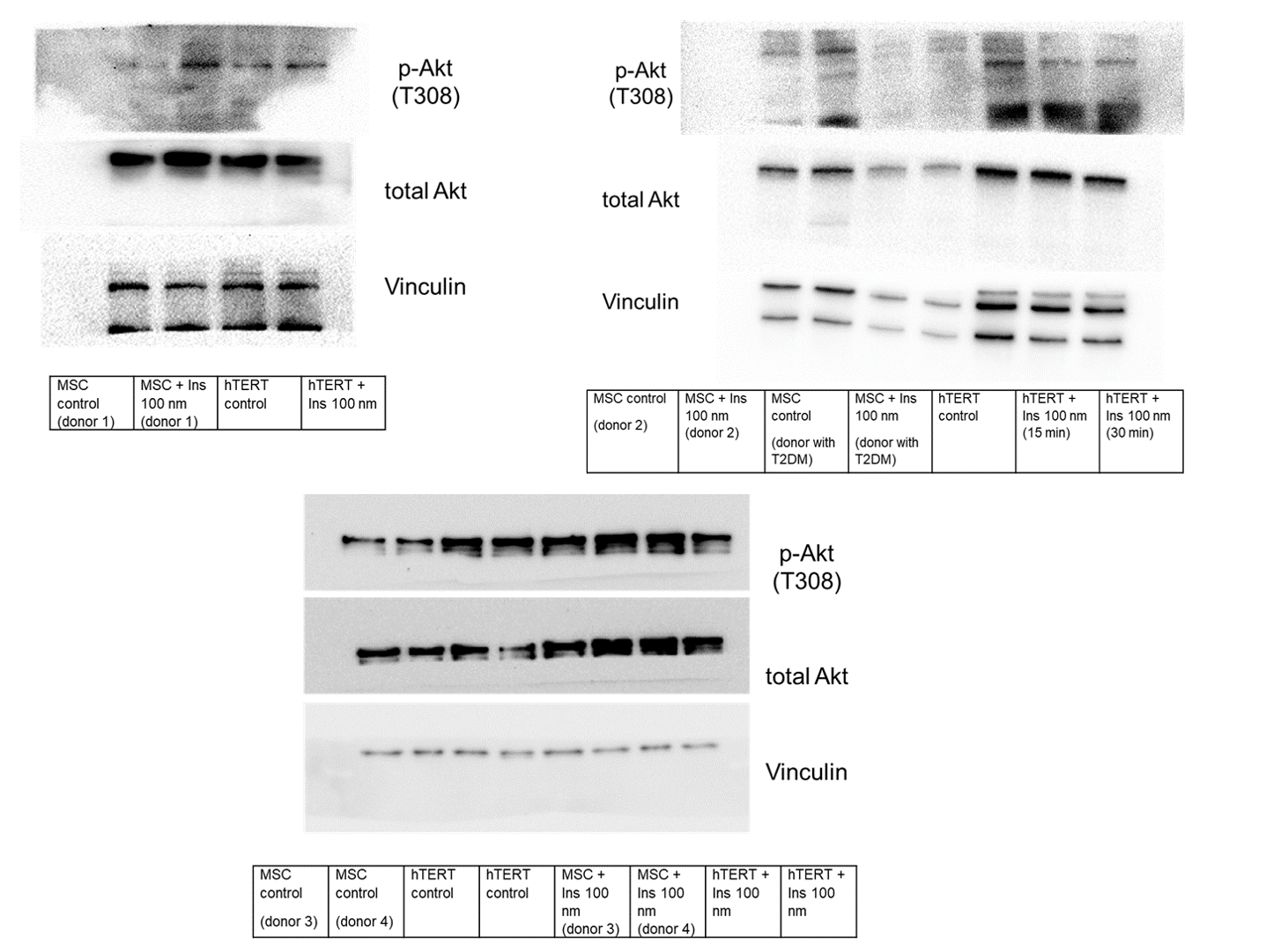

Supplement: Supplementary file 2 [file Image_2.TIF]
